# 2 Examination of Burn Resuscitation Complications from the Burn Navigator Observational Trial

**DOI:** 10.1093/jbcr/irac012.006

**Published:** 2022-03-23

**Authors:** Julie A Rizzo, Elsa Coates, Jose Salinas, Maria Serio-Melvin, Tam N Pham, Kareem R Abdelfattah, Kevin N Foster, Nehemiah T Liu

**Affiliations:** US Army Institute of Surgical Research, Fort Sam Houston, Texas; US Army Institute of Surgical Research, JBSA Fort Sam Houston, Texas; US Army Institute of Surgical Research, San Antonio, Texas; US Army Institute of Surgical Research, Cibolo, Texas; University of Washington, Seattle, Washington; UT-Southwestern Medical Center, Dallas, Texas; The Arizona Burn Center Valleywise Health, Phoenix, Arizona; U.S. Army Institute of Surgical Research, San Antonio, Texas

## Abstract

**Introduction:**

Burn care continues to focus on providing enough fluid resuscitation to perfuse end organs with the least amount of fluid necessary in order to prevent complications related to excess fluid. In this observational trial of 5 ABA-verified burn centers that utilized the Burn Navigator (BN), a clinical decision support tool, we sought to examine resuscitation-related complications that occurred in the first 48 hours after burn injury. Since minimal literature exists regarding the incidence of resuscitation-related complications in the acute phase after burn injury, we aimed to present our data for future comparison.

**Methods:**

An observational study of adult patients undergoing burn resuscitation utilizing the BN was conducted. Data were gathered hourly for the first 48 hours for patients on fluid infusion rates, laboratory data, critical care elements to include ventilator settings and clinically relevant outcomes. Morbidities were classified based on each burn center’s definition as related to over or under-resuscitation and variables associated with these outcomes were extracted from the data set.

**Results:**

Three hundred patients were enrolled into the study, and 156 resuscitation-related complications were documented in 92 patients in the first 48 hours after admission. Compartment syndromes (abdominal, extremity, ocular) accounted for 62 (40%) of the complications. ARDS occurred in 9 patients. ARDS patients were the most severely injured, reflected by highest Baux score. None of the ARDS patients had an inhalation injury. The under-resuscitation morbidities of shock and acute kidney injury accounted for 81 (52%) of the complications. Patients experiencing shock received greater than the Parkland formula in the first 24 hours after injury. Most patients with AKI continued to make adequate urine during their resuscitation period, with 59% making an average of >30 ml/hr over the first 24 hours. Nearly half of patients with AKI were placed on renal replacement therapy in the first 48 hours. Seventeen patients (18.5%) experienced both a compartment syndrome and either AKI or shock.

**Conclusions:**

This large observational study demonstrates variables associated with different complications across 5 major burn centers and shows that complications associated with over- and under-resuscitation can occur within the same patient during resuscitation after burn injury. Additional comparative studies are needed to better understand the cause of these complications, to determine the incidence of these complications in a larger population and criteria used to define each complication.

